# ECG Parameters for Malignant Ventricular Arrhythmias: A Comprehensive Review

**DOI:** 10.1007/s40846-017-0281-x

**Published:** 2017-06-28

**Authors:** Satria Mandala, Tham Cai Di

**Affiliations:** 1 Fakultas Informatika, Universitas Telkom, Jl. Telekomunikasi Terusan Buah Batu, Bandung, 40257 Indonesia; 2 IJN-UTM Cardiovascular Engineering Centre, Universiti Teknologi Malaysia, Johor, Malaysia

**Keywords:** Ventricular arrhythmia, Prediction, ECG parameter, Behavior pattern

## Abstract

Many studies showed electrocardiogram (ECG) parameters are useful for predicting fatal ventricular arrhythmias (VAs). However, the studies have several shortcomings. Firstly, all studies lack of effective way to present behavior of various ECG parameters prior to the occurrence of the VAs. Secondly, they also lack of discussion on how to consider the parameters as abnormal. Thirdly, the reports do not include approaches to increase the detection accuracy for the abnormal patterns. The purpose of this study is to address the aforementioned issues. It identifies ten ECG parameters from various sources and then presents a review based on the identified parameters. From the review, it has been found that the increased risk of VAs can be represented by presence and certain abnormal range of the parameters. The variation of parameters range could be influenced by either gender or age. This study also has discovered the facts that averaging, outliers elimination and morphology detection algorithms can contribute to the detection accuracy.

## Introduction

Malignant spontaneous ventricular arrhythmias (VAs), namely ventricular tachycardia (VT) and ventricular fibrillation (VF) can cause sudden cardiac arrest. Patients who are susceptible to VT/VF always have a risk of sudden cardiac death [1]. Therefore, prediction of VT/VF prior to their initiation is vital to avoid delay of rescue actions [2].

Researchers has discovered that ECG signals comprise non-invasive parameters that could reflect underlying VT/VF [3–5]. The ECG parameters, such as fragmented QRS (fQRS), heart rate variability (HRV), T peak-T end (TpTe), heart rate turbulence (HRT) and T wave alternans (TWA) have predictive value for the arrhythmic events [6–8]. In this case, cardiac monitoring by analyzing the ECG parameters is an effective way to determine future occurrence of the fatal VT/VF.

In order to track development of the ECG parameters as promising predictors, a comprehensive review is necessary. A few reports, such in [3, 4] seem can be considered falls into this area. However, they lack of effective way to present pattern of various ECG parameters prior to the occurrence of the VAs. It is hard to distinguish the patterns that represent increased risk of the arrhythmias from these detailed reviews [3, 4]. This is because of authors of the reviews tend to introduce the identified parameters without grouping their similarity in patterns prior to the arrhythmias.

Second, the reports are also not clearly in defining how to consider the patterns as abnormal. For example, researchers in [3] only present a hazard ratio for sudden death due to abnormal QT prolongation, but not range of the parameters that are considered as abnormal. In this case, the researchers have to find out certain values that can represent the abnormality via either experiments or other literatures. This process is scientifically proven time-consuming.

Third, these reports have not included approaches to increase detection accuracy of the parameters. The detection accuracy is important because the abnormal patterns may undetected if the parameters fail to be accurately identified. A report that is presented in [4] stated conflicting results of prolonged QT interval (QT_i_) as predictors when there are difficulties in measuring the QT_i_ correctly. The discussed problems so far leave three important questions unanswered:

i. How to effectively present the patterns of the ECG parameters prior to occurrence of the VAs?
ii. How to identify abnormal patterns for the parameters?
iii. How to improve detection accuracy of the ECG parameters?

These problems are selected to be solved since the solutions are beneficial in two aspects. Firstly, they could speed up the process of reviewing the parameters. It is because the brief information of the parameters will be firstly presented in a table before further discussion. The solutions simplify relevant information for the ECG parameters and thus researchers can have a clear idea of the parameters. Secondly, they provide a way to improve quality of researches. The researches that have unsatisfied prediction result can apply the solutions in the review to produce better outcome.

To address above the problems, this article covers results of works as follows:

i. This paper groups the patterns for the increased risk of the arrhythmias based on behavior changes of these ECG parameters before the arrhythmias. Some parameters are absent in normal condition of cardiac patients, and only present before the VAs onset. There are also parameters that indicate the future VAs if prolong or decrease until certain ranges. The behavior changes could be increase, decrease or presence. The groups of the parameters will be further discussed in details in Sect. 3.
ii. This paper describes abnormal behaviors of the parameters according to their normal ranges. As aforementioned, the parameters, which have value either higher or lower than certain range, have been considered as increased risk for the arrhythmic events. For instance, patients with TpTe greater than 100 ms have increased risk of the VT/VF [7–9]. Gender could be a factor that affects the abnormal range for the parameters, such as HRV and QT_i_ [10, 11].
iii. The parameters are mostly derived from two ECG components, namely QRS duration and T-wave. This paper has identified several suggested methods from literatures to increase correct detection rate of the parameters used in the VAs prediction.

This paper consists of six sections. It begins with an Introduction section, followed by four sections that describe ECG parameters that can predict the life-threatening VT/VF, and ends with a Conclusion section. The first section explains selection of the potential ECG parameters. The next two sections are inline with the identified three problems. And, the fourth section provides a discussion on the selected ten ECG parameters that have predictive value for occurrence of VAs.

## Identification and Selection of ECG Parameters that have Predictive Value for VA Onset

**Fig. 1:**
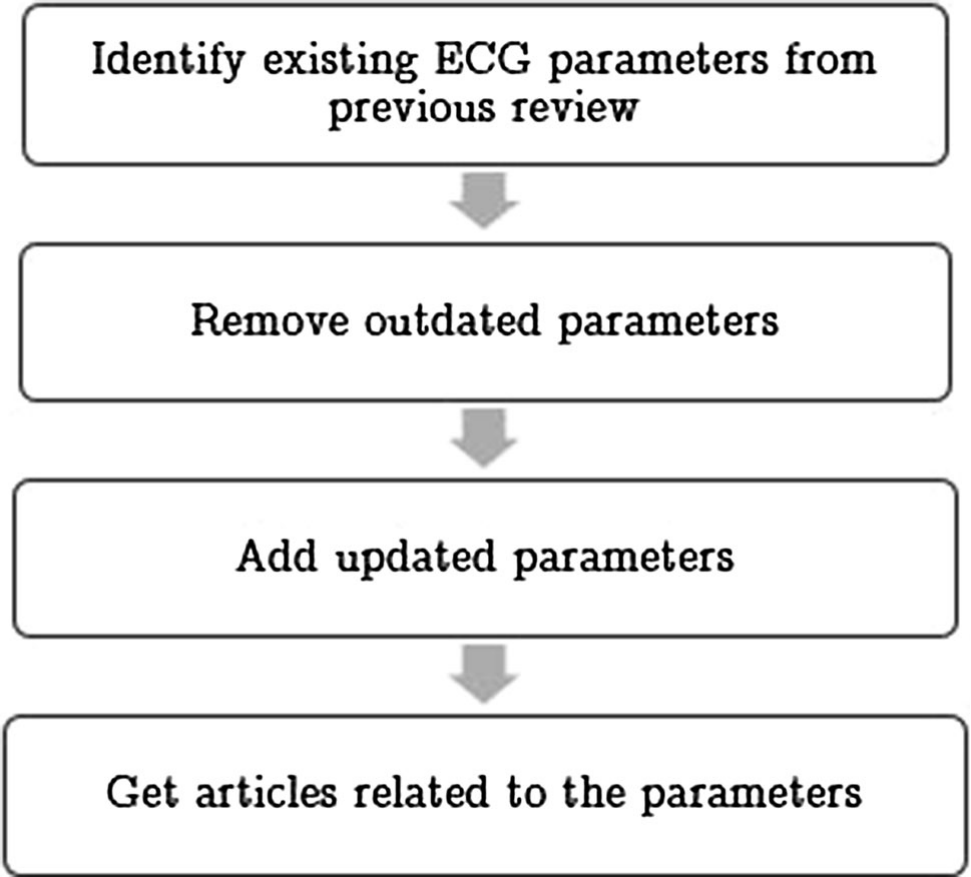
Flow chart of identifying existing ECG parameters

There are many ECG parameters that have been reviewed before, such as early repolarization, QT_i_/QT dispersion, signal-averaged ECG, and HRV [3, 4]. However, these reviews still have disadvantages as stated in Sect. 1. Moreover, there might have other new potential parameters in the recent years. In this case, this paper will select the source articles for review using approaches as shown in Fig. 1.

As illustrated in Fig. 1, ECG parameters to be reviewed are firstly identified based on existing reviews [3, 4]. This is because some of the parameters in the reviews are still relevant and reliable in recent studies. Moreover, to increase reliability of this paper, the parameters that are lack of related researches in last 10 years are removed. In addition, the potential parameters that are newly discovered in recent 5 years will be included in the review. Based on the aforementioned selection, ten parameters from 25 articles will be included for review, as shown in Table 1.

**Table 1:** Overview of ECG parameters

ECG parameters	Derived from	Morphology based/measurable	Prediction of VAs for patients with certain diseases
QT_c_/QT_v_i/QT_v_i/QTd [8, 23, 24]	QT interval	Measurable	Recurrence of VAs, EF, structural heart disease, AMI
fQRS [18–21]	QRS duration	Morphology based	ARVCM, HOCM, AMI, IDCM
ER [16, 22]	QRS duration	Morphology based	recurrence of VAs, CAD
VLP [14, 15, 17]	QRS duration-ST segment	Morphology based	BS, STEMI
HRV [28, 29]	RR interval	Measurable	AMI
iCEB [30, 31]	QT interval and QRS duration	Measurable	LQTS, BS
QT dynamicity [26, 27]	QT interval and RR interval	Measurable	AMI, IDCM
HRT [6, 25]	RR interval	Measurable	AMI
TWA [6, 25]	T wave/ST segment	Morphology based	AMI
TpTe [7–9]	T wave	Measurable	AMI, recurrence of VAs, Cha- Gas

*AMI* acute myocardial infarction, *BS* Brugada syndrome, *ARVCM* arrhythmogenic right ventricular cardiomyopathy, *CAD* coronary artery disease, *EF* ejection fraction, *HOCM* hypertrophic obstructive cardiomyopathy, *IDCM* idiopathic dilated cardiomyopathy, *LQTS* long QT syndrome, *STEMI* acute ST-segment elevation myocardial infarction

**Fig. 2:**
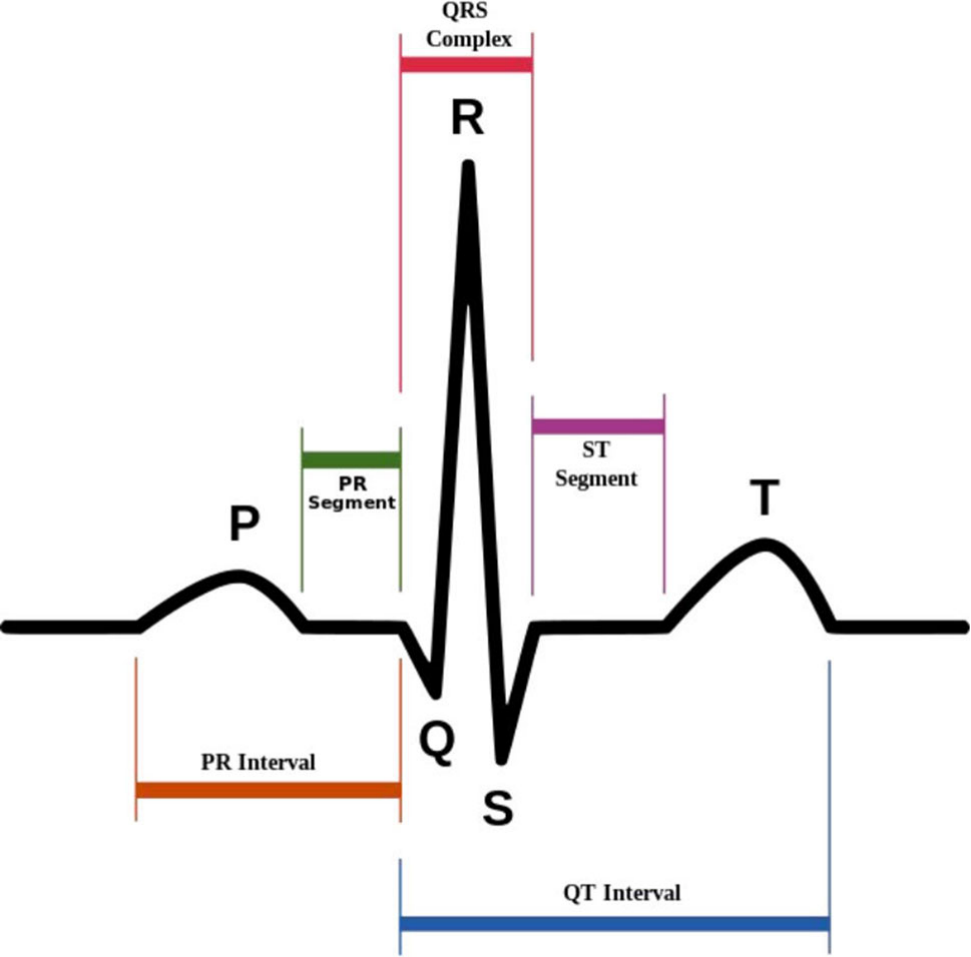
Components of normal ECG signal

Table 1 presents an overview for the ten parameters. The first column in the table lists the identified ECG parameters. The next column related to derivation of the parameters from ECG signal components. The components of a normal ECG signal are as illustrated in Fig. 2. From the components of the ECG signals, the parameters are used in predicting future arrhythmic event of a patient via morphology based algorithms or measurable values, as shown in third column. The last column states the prediction of the arrhythmias are successful in patients with certain diseases.

As it can be seen from the Table, the parameters are derived from different ECG signal components, such as QRS complex, which can be used for deriving fQRS, ER and VLP. In addition, based on methods to identify parameters, the parameters are categorized into morphology based and measurable. QT_c_/QT_v_i/QT_v_i/QT_d_, HRV, HRT, iCEB, QT dynamicity and TpTe are parameters that can be easily measured if relevant ECG components are detected. In some cases, the parameters might have different predictive result in patients with different diseases. Therefore, the parameters are tested for various diseases. The details are further discussed in the following paragraphs.

As shown in Table 1, the parameters are derived from different ECG signal components, such as QRS complex, which can be used to derive fQRS, ER and VLP. In addition, based on methods to identify parameters, the parameters are categorized into morphology based and measurable. QT_c_/QT_v_i/QT_v_i/QT_d_, HRV, HRT, iCEB, QT dynamicity and TpTe are parameters that can be easily measured if relevant ECG components are detected. In some cases, the parameters might have different predictive result in patients with different diseases. Therefore, the parameters are tested for various diseases. The details are further discussed in the following paragraphs.

**Fig. 3:**
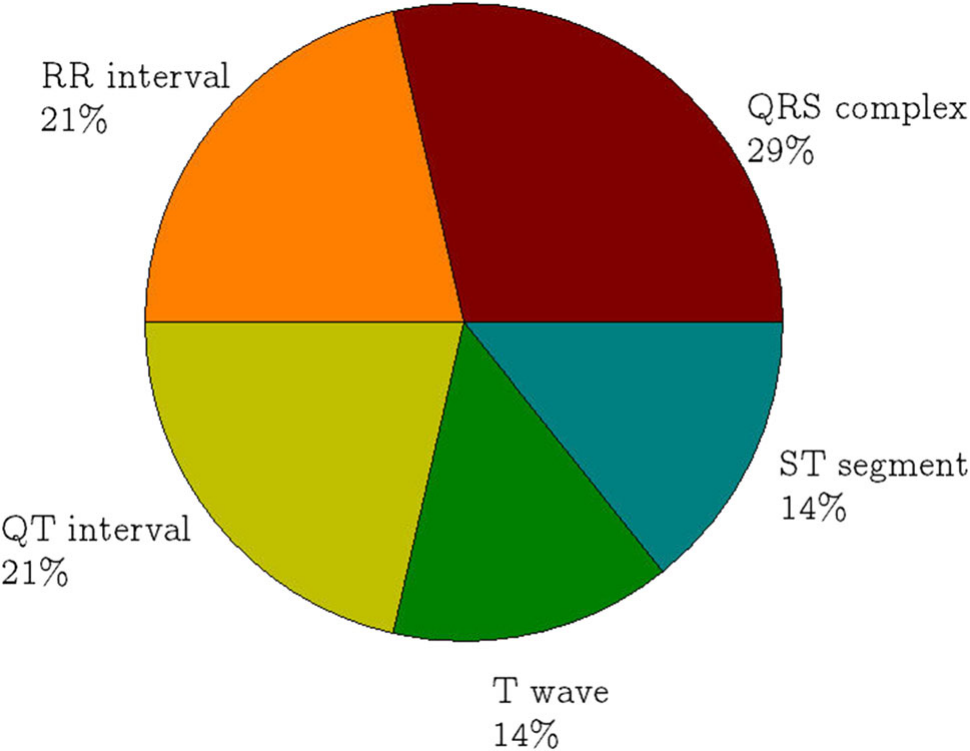
Derivation of the ECG parameters

Figure 3 shows that the ten parameters in Table 1 are derived from six different components of the ECG signal. 64% of the parameters can be derived from QRS duration, RR interval (RR_i_) and T wave; the rest are derived from QT interval and ST segment. The former components are easier to be acquired since they only involve detection of one component of the signal, i.e. QRS complex, R wave or T wave. Thus, most of the parameters, such as fQRS, HRV and TpTe are derived from these components. Whereas, the latter components require detection of both QRS complex and T wave to derive the parameters, where failure detection in either one component can cause the derivation unsuccessful.

**Fig. 4:**
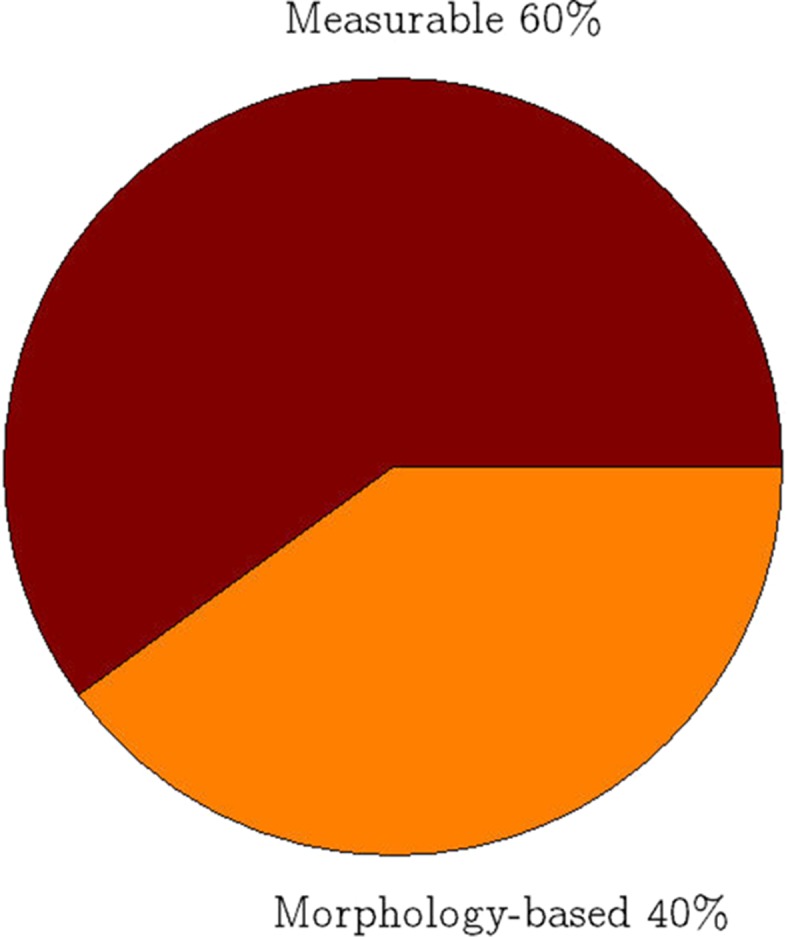
Morphology based and measurable ECG parameters

Figure 4 illustrates the differences of the parameters in pie chart based on methods to identify parameters, which can be categorized into two groups, i.e. morphology based parameters and measurable parameters. Both occupy 40 and 60% of the pie chart respectively. The morphology based parameters, such as fQRS and ER are based on morphology detection algorithms to include all of their possible variations [12, 13]. The morphology parameters occupy smaller percentages because their detection have to consider various morphologies of the parameters. Whereas, the measurable parameters, such as HRV and QT interval related parameters are measured after detection of the ECG components. Using the value, abnormality of the parameters can be determined.

**Fig. 5:**
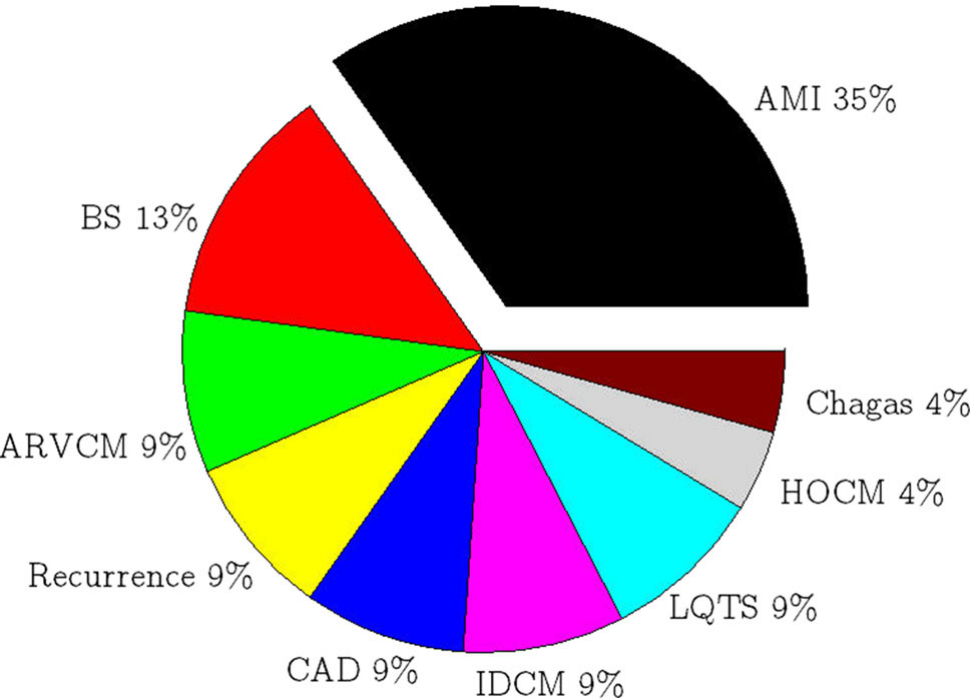
Heart diseases and ECG parameters

The ECG parameters are useful in predicting the VAs for patients with certain diseases. As shown in Fig. 5, 35% studies assess predictive value of the parameters in patients with acute myocardial infarction (AMI). This is because of the arrhythmic events are usually occur during the natural course of AMI [7]. In addition, the parameters [14, 15] are also prevalent in patients with Brugada syndrome (BS), which occupy 13% of the studies in total. The parameters are less frequent in patients with other diseases, such as arrhythmogenic right ventricular cardiomyopathy (ARVCM) [15], recurrence of the VAs [8] and coronary artery disease (CAD) [16]. Thus, there are fewer relevant studies.

## The Ten Potential ECG Parameters

Section 2 identifies ten ECG parameters that have potential values to demonstrate underlying ventricular arrhythmias. These parameters are heart rate corrected QT interval (QT_c_), QT interval variability (QT_v_i), QT interval dispersion (QT_d_), fQRS, early repolarization (ER), ventricular late potentials (VLP), HRV, HRT, index of cardio electro physiological balance (iCEB), QT dynamicity, TWA as well as TpTe. The parameters have various behavior changes prior to VA onset.

Table 2 shows the changes of the parameters prior to the VAs onset can be grouped into three behaviors, i.e. presence, increase and decrease. The presence of three ECG parameters, namely VLP [14, 15, 17], fQRS [18–21] and ER [16, 22] could be associated with an increased incidence of ventricular arrhythmic events. These three parameters appear precede the arrhythmic event. As listed in Table 2, it is also found that the increased risk of the VT/VF occurrence can be represented by either prolongation or reduction of the parameters, based on certain abnormal range. The increase or prolongation value of the parameters, including TpTe [7–9], heart rate corrected QT interval (QT_c_) [8] /QT_v_i [23] /QT_d_ [8, 24], turbulence onset (TO) of HRT [25], QT dynamicity [26, 27], as well as TWA [6, 25] might indicate higher risk for the arrhythmias. Table 2 also reveals that the increased risk of the arrhythmias can also be represented by decrease or reduction in ECG parameters, such as HRV [28, 29] and turbulence slope (TS) of HRT [6, 25]. The iCEB [30, 31] is the most potential predictor for predicting an increased susceptibility to torsades de pointes (TdP) or non-TdP VT/VF based on its increase or decrease value.

**Table 2:** ECG parameters and their behaviours prior to ventricular arrhythmias

ECG parameter	Behavior		
	Presence	Increase/prolongation	Decrease
QT_c_/QT_v_i/QT_d_ fQRS		✓	
ER	✓		
VLP	✓		
HRV	✓		✓
iCEB		✓	✓
QT dynamicity		✓	
HRT		✓ (TO)	✓ (TS)
TWA		✓	
TpTe		✓	

The increase or decrease of the parameters can be identified according to certain ranges. In reviewed literatures, researchers utilized two methods to define abnormal ranges. Firstly, the researchers match the parameters with predefined threshold values that indicate the abnormal ranges. The parameters that have abnormal prolongation prior to the arrhythmias are iCEB, QT dynamicity, QT_c_/QT_v_i/QT_d_, TO of HRT, TWA and TpTe. Whereas, the parameters that have lower values than normal heart condition are HRV and TS of HRT. The parameters, such as fQRS, VLP and ER, in which their presence indicate the increased risk of VAs can also be identified using predefined thresholds. The details of the parameters are discussed further in the following subsections. Secondly, the researchers compare the parameters in two groups, namely healthy people and patients prone to the arrhythmias [28]. Using extracted values from these two groups, the researchers should identify threshold that represents higher risk of VAs occurrence. This method may be more suitable for group of study subjects that have no gender and age control. This is because of gender and age could be two factors that affect the abnormal ranges.

In order to know whether the parameters are beneficial in clinical practice to guide decision making, a cutoff for predicted probability is needed. An optimal cutoff is defined by decision context. Once the cutoff is selected, clinical usefulness measures can be defined [32]. The measures used in reviewed articles that listed in Table 1 are P value (P), accuracy (Ac), sensitivity (Se), specificity (Sp), positive predictive value (+P) and area under receiver operating characteristic curve (ROC-AUC). According to the reviewed literatures, P value is the most commonly used measure. The P value is a probability of obtaining an observed result, plus more extreme result, assuming the truth of the null hypothesis. Statistically significant P value (e.g. P < 0.05) is not informative about data that are analyzing, i.e. the data are unlikely with a true null hypothesis [33]. This provides substantial evidence that the null hypothesis can be rejected. Whereas, ROC-AUC ranged from 0 to 1. An area of 1 represents a perfect test; an area of 0.5 represents a worthless test [6].

In addition, Ac is a ratio of total correct assessments to all assessments, where Ac = (TN + TP)/(TN + TP + FN + FP). Next, Se is related to an ability to correctly identify patients with VT/VF risk, where Se = TP/(TP + FN). Sp is related to an ability to correctly identify either normal people or patients with VT/VF risk, where Sp = TN/(TN + FP). And, +P is a probability where a patient with positive test is actually at risk of VT/VF, where +P = TP/(TP + FP). These four measures represent a perfect test if 100% is obtained. Using the aforementioned clinical usefulness measures, the ten potential ECG parameters are discussed together with their behavior changes preceding VAs onset in the following subsections.

### Ventricular Late Potentials (VLP)

**Fig. 6:**
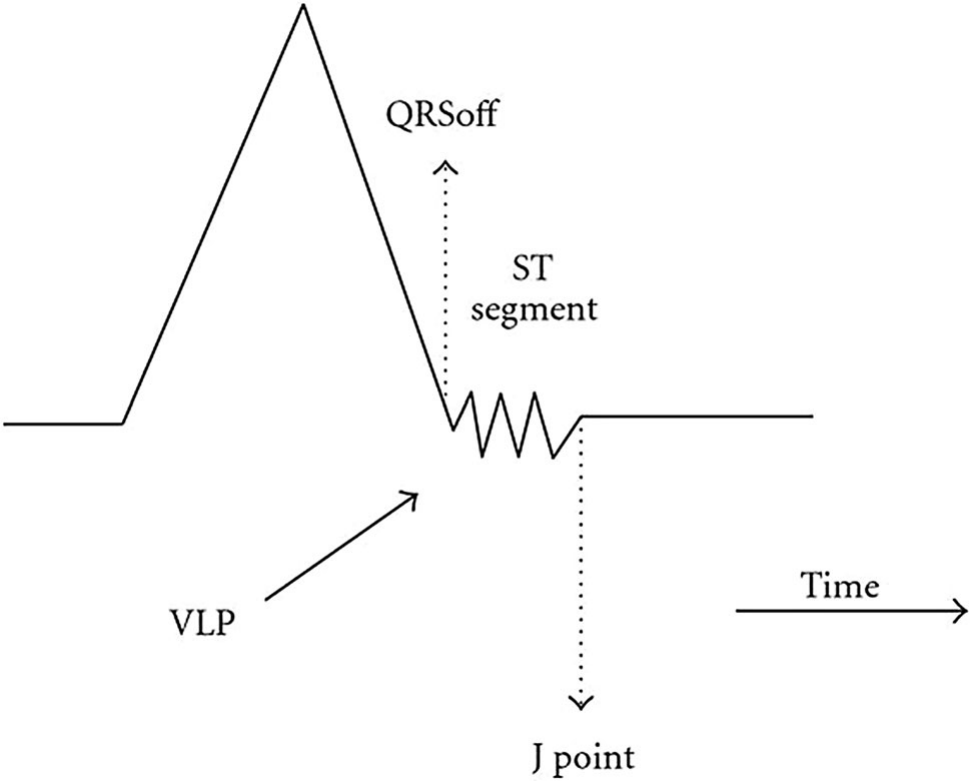
Morphologies of VLP *Source* [34]

The VLP is a high-frequency and very-low-intensity signal that is localized at the end of QRS duration, and on the beginning part of ST segment [34], as shown in Fig. 6.

The presence of the VLPs can be identified using predefined thresholds. According to studies [17, 35], the VLPs exist if three conditions of a filtered QRS duration are met. The first condition is the filtered QRS duration is at least 114 ms. And, the filtered QRS duration that has low amplitude signal (<40 µV) in the terminal portion of the filtered QRS duration of at least 38 ms. The last condition is a root mean square of at most 20 µV in the terminal portion of the filtered QRS duration of 40 ms.

The VLP that is detected from signal-averaged electrocardiogram (SAECG) system could be a useful VT/VF predictor for BS and STEMI patients. The VLP has been obtained from 24 h ECG monitoring produced a P value of 0.003 in a recent BS study [15]. This indicates that assuming the VLP has no predictive value for BS patients, an observed result or more extreme result is obtained in 0.3% of experiments. Moreover, another study [17] showed that assuming the VLP has no predictive value for STEMI patients, an observed result or more extreme result is achieved in less than 5% of experiment.

### Fragmented QRS (fQRS)

**Fig. 7:**
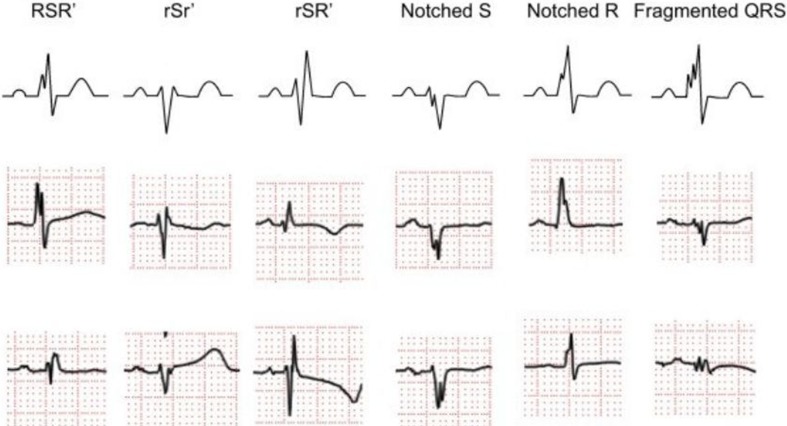
Morphologies of fragmented QRS *Source* [36]

The fQRS is additional spikes within QRS duration without bundle branch block [36], which can possess several morphologies, such as additional R wave, notched R or notched S wave [5, 37], as illustrated in Fig. 7.

In a recent study [12], researchers proposed an automated algorithm based on several thresholds for detection the various morphologies of fQRS. The algorithm has two important modules in which the details are discussed in the relevant literature, namely fragmentation detection and morphology identification. The fragmentation detection module uses several rules for identifying extrema and notches based on detailed discrete wavelet transform (DWT) coefficients of QRS duration. On the other hand, the morphology identification module encompasses recognization of six fundamental morphologies of fQRS and other variations of RSR’ patterns.

The fQRS is a significant predictor for patients with ARVCM [18], HOCM [19] and IDCM [21]. The fQRS on 12-lead ECG produced P < 0.001 and P < 0.05 in Canpolat et al. [18] and Femenia et al. [19] studies respectively. If the fQRS has predictive value for IDCM patients, an observed result or more extreme result is obtained in 95.5% of experiments [21].

### Early Repolarisation (ER)

**Fig. 8:**
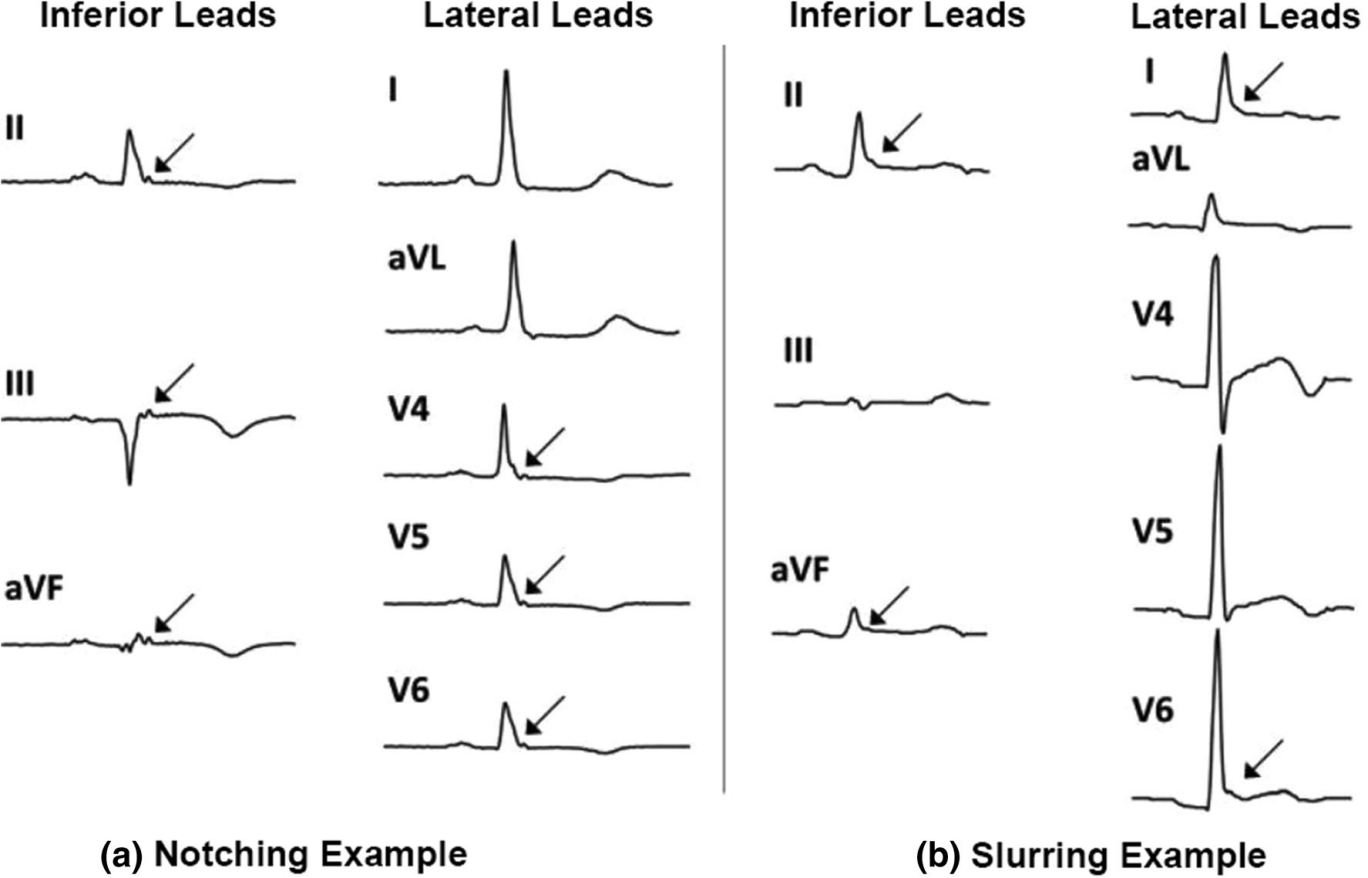
Morphologies of early repolarization

Similar to both VLP and fQRS, the ER can also be detected at QRS duration. ER is a notching or slurring morphology of the terminal QRS in at least two contiguous inferior or lateral leads [16, 38, 39], as depicted in Fig. 8. ER can be benign or malignant. In general, malignant ER is associated with older age, a significantly longer QRS duration and increased sign of VAs [40]. A prominent J wave is a noticeable finding just before the VA onset [22, 41]. In addition, a horizontal or descending ST segment is also often emphasized in reports of malignant ER [40, 42].

The notched ER and slurred ER patterns can be determined using an algorithm as shown in Kentta et al. study [43]. The algorithm analyzes ECG leads independently and classifies each lead as notched, discrete, slurred, or negative. In addition to these four categories, a fifth category, indeterminate is used if the morphology of a lead cannot be definitely classified by the algorithm.

The ER is associated with increased risk of fatal VT/VF in patients with CAD [16] and VF history [22]. Based on Patel et al. study [16], ER in 12-lead ECG has P = 0.005 and P = 0.03 in inferior leads (leads II, III, and a VF). The ER is more common in inferior leads compared to other leads. The P value of the ER for recurrent VF is 0.008 [22]. Assuming the ER has no predictive value for recurrent VF patients, an observed result or more extreme result is achieved in 0.8% of experiment.

### T peak-T end (TpTe)

The TpTe is defined as time interval between T wave peak amplitude and the end of T wave [44. TpTe can be a useful predictor for patients with diseases, such as Chagas disease [9], Brugada syndrome [44], and myocardial infarction [7, 45].

The definition of abnormal prolongation for TpTe is value larger than 100 ms [7–9]. The prolonged TpTe in 12-leads ECG and implantable cardioverter defibrillators (ICD) indicates VT/VF risk in patients with AMI [7], VAs history [8] and Chagas disease [9]. These three patient groups have P value of less than 0.01, 0.006 and 0.07.

### QT Interval Related Parameters (QT)

The QT_i_ is measured from the start of Q wave to the end of T wave [46]. QT_c_ is QT_i_ corrected for heart rate [8, 46]. QT_d_ is difference between maximum and minimum of QT_i_ [24]. And, QT_v_i is a ratio comparing repolarization variability to HRV [47]. Equations for the QT_i_ related parameters are as shown in Eqs. 1, 2, and 3.

1 $$QT_{c} (Bazettformula) = QT_{i} x\surd \left( {1/RR} \right)$$

where RR_i_ is measured from one R wave to the next R wave before the QT_i_

2 $$QT_{d} = QT_{max} - QT_{min}$$

3 $$QT_{v} i = log(\left( {varianceQT_{i} /meanQT^{2} } \right)/\left( {varianceRR/meanRR^{2} } \right))$$

The abnormal ranges for QT_c_, QT_d_ and QT_v_i are values greater than 460 ms, 65 ms and −0.47 respectively [8, 48]. According to Cox [10], the normal QT_i_ usually ranged from 0.36 to 0.44 s. From these ranges, adult males have shorter range than adult females, and people from age 1 to 15 are in between them, as shown in Table 3. According to the table, QT_i_ with value greater than 0.47 s is considered as dangerous for any gender or age.

**Table 3:** Normal QTi ranges *Source* [10]

	Age 1–15	Adult man	Adult woman
Normal	<0.44 s	<0.43 s	<0.45 s
Borderline	0.44–0.46 s	0.43–0.45 s	0.45–0.47 s
Prolonged	>0.46 s	>0.45 s	>0.47 s

*s* second

Measuring of the QT related parameters can detect patients who are at increased risk of developing VT/VF and with diseases such as structural heart disease [23], AMI [24] and VAs history [8]. If the QT_v_i on ICD has predictive value for patients with structural heart disease, an observed result or more extreme is obtained in more than 96% of experiments [21]. Assuming the QT_i_ has no predictive value for patients with no recurrence of VAs and AMI, an observed result or more extreme result is achieved in less than 1.1 and 0.1% of experiments respectively [8, 24].

### T Wave Alternans (TWA) and Heart Rate Turbulence (HRT)

**Fig. 9:**
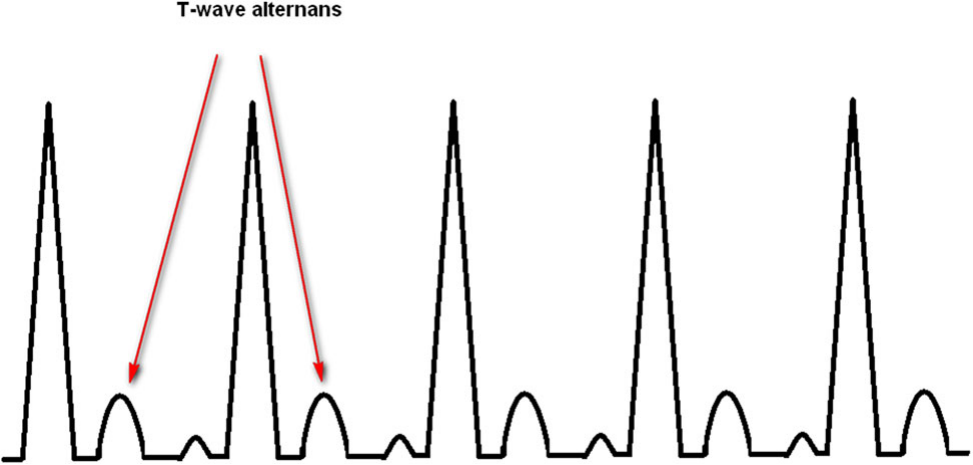
Measurement of TWA

The TWA is a repeating ABAB pattern in the morphology and amplitude of T wave or ST segment [49], as illustrated in Fig. 9. It reflects a continuum of cardiac electrical instability [50].

The TWA can be either an independent predictor [51] using Eq. 4 or a combined predictor with HRT [6, 25]. However, TWA might not be a useful predictor for exercising individuals since it can be occurred in normal individuals at heart rates more than 120 beats/min [52].

4 $$Kscore = \frac{{P_{0.5} - \mu }}{\sigma }$$

where *μ* and *σ* are mean and standard deviation of spectral noise, *P*_0.5_ is alternans power at 0.5 cycles/beats and (*P*_0.5_ − *μ*) is alternans voltage. The TWA is considered as significant if K score is more than three [53].

The HRT is a short-term fluctuation in sinus cycle length that follows spontaneous ventricular premature complex, in which consists of brief heart rate acceleration, followed by more gradual heart rate deceleration [54]. The acceleration is quantified by TO. The TO can be represented as Eq. 5.

5 $$TO = \frac{{\left( {RR_{1} + RR_{2} } \right) - \left( {RR_{ - 2} + RR_{ - 1} } \right)}}{{RR_{ - 2} + RR_{ - 1} }}\times100\%$$

where RR_−2_ and RR_−1_ are two intervals immediately preceding ventricular pre- mature complexes (VPC); and RR1 and RR2 are two RR intervals immediately after compensatory pause.

The another parameter derived from HRT is TS, which represents the deceleration of heart rate. The TS can be measured based on the maximum positive regression slope assessed over any five consecutive sinus rhythm RR interval within the first 15 sinus rhythm RR intervals after ventricular premature contraction [25]. Therefore, the TO can be defined as measure of relative shortening of RR after pause of ventricular premature beat. Whereas, the TS characterizes subsequent lengthening of RR [6].

Both HRT and TWA have different thresholds to identify abnormal behavior. The abnormal TO of HRT in patients have value 0% and above. On the other hand, the abnormal TS of HRT is value less than 2.5 ms per RR_i_ [25, 54]. And, the abnormal prolongation of TWA is value more than 46 µV [6, 25].

The combined TWA and HRT on 12-leads ECG and implantable cardioverter defibrillators (ICD) indicates VT/VF risk in patients with AMI [6, 25]. According to Arisha et al. study [6], a combined TO of HRT and TWA on channel 1 has strong predictive performance for VT/VF, where ROC- AUC = 0.8, P = 0.03 Se = 80% and Sp = 79%. In addition, both abnormal TWA and HRT have achieved P value 0.002 [25].

### Heart Rate Variability (HRV)

The HRV has both time domain and frequency domain variables that have predictive power for the arrhythmic events. Examples of the time domain variables are standard deviation of all RR/normal-to-normal (NN) intervals (SDNN) and mean of RR/NN interval (RRm) [28], which can be expressed as Eqs. 6 and 7; examples of the frequency domain features are low frequency (LF) and high frequency (HF) components [28, 55].

6 $$RR_{mean} = 1/n\mathop \sum \nolimits RR_{i}$$

7 $$SDNN = \sqrt {1/n\mathop \sum \nolimits (RR_{i} - RR_{mean} )^{2} }$$

The abnormal HRV can be recognized by comparing both normal range of HRV in healthy people and abnormal range in patients prone to the arrhythmias. In average, normal range of HRV for females is low than males [56]. Statistics for the normal ranges of HRV parameters are as illustrated in Table 4. Comparison of both time domain and frequency domain parameters amongst males and females showed attenuated HRV in females. Among the HRV parameters, SDNN_i_ and LF are found significantly decreased in females, i.e. P < 0.05.

**Table 4:** Comparison of HRV parameters between male and female subjects *Source* [56]

HRV parameters	Group of individuals		P value
	Males	Females	
Time domain (ms)			
SDNN	140 ± 36	122 ± 33	0.09
SDANN	123 ± 34	111 ± 34	0.23
SDNN_i_	64 ± 19	52 ± 14	0.03
rMSSD	40 ± 14	40 ± 22	0.9
pNN50	14 ± 10	12 ± 7	0.43
Frequency domain (ms^2^)			
Total power	4041 ± 3150	2750 ± 1493	0.07
VLF	2912 ± 2675	1843 ± 928	0.06
LF	788 ± 397	556 ± 346	0.04
HF	318 ± 251	312 ± 277	0.94

*SDNN* standard deviation of RR intervals, *SDANN* standard deviation of average NN intervals, *SDNN*_*i*_ SDNN index, a measure of variability due to cycles shorter than 5 min, *rMSSD* square root of the mean squared differences of successive NN intervals, *pNN50* number of interval differences of successive NN intervals greater than 50 ms, NN50/total number of NN intervals, *VLF* very low frequency, *LF* low frequency, *HF* high frequency

In this review, the HRV is the only one predictor that is used in short term VT/VF prediction. Ebrahimzadeh et al. [28] shows time–frequency and non-linear features of HRV on 24 h ECG can predict the VT/VF that cause sudden cardiac death. The prediction time is four minutes prior to its occurrence, with accuracy more than 83%. Rozen et al. study [29] also reveals that HRV is predictor for imminent VT with 50% Se, 91.6% Sp and 84.5% +P.

### Index of Cardiac Electrophysiological Balance (iCEB)

**Fig. 10:**
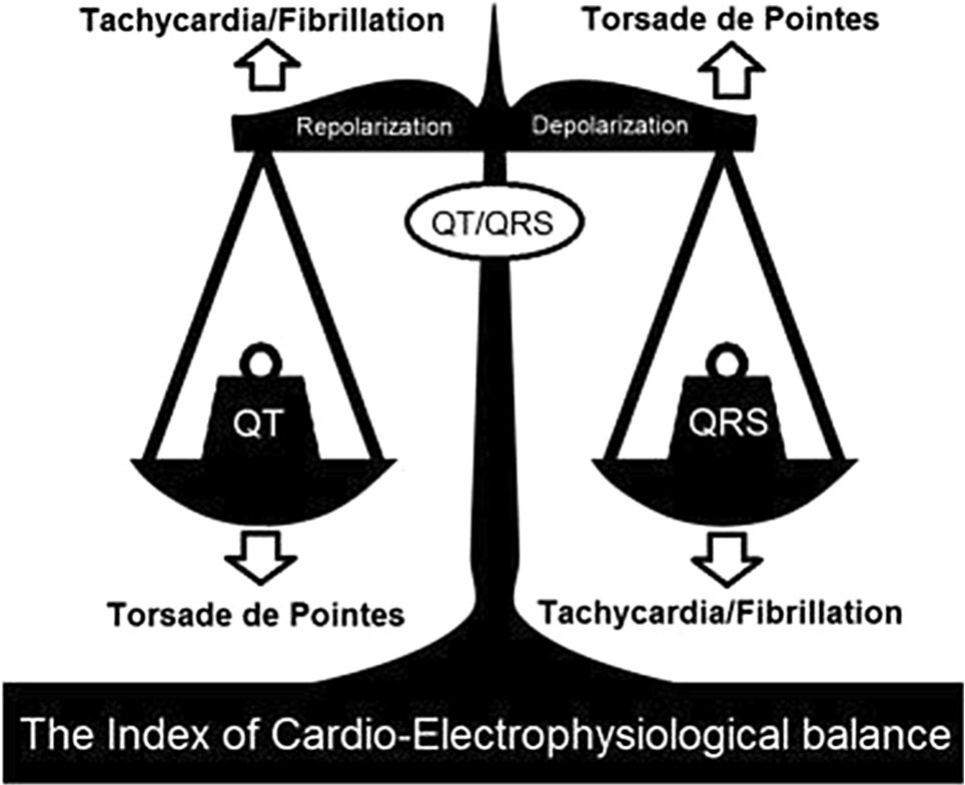
Measurement of iCEB *Source* [31]

The iCEB is a hypothesized that equivalent to cardiac wavelength, which is a product of effective refractory period and conduction velocity (ERP × CV). It reflects balance and imbalance of depolarization (QRS duration) and repolarization (QTi) of the cardiac electrophysiology, as shown in Fig. 10 [30, 31]. The iCEB can be represented as in Eq. (8). Lu HR et al. [31] enhanced their previous study [30], which investigated predictive value of iCEB in both TdP and nonTdP mediated VT/VF. Based on [31] study, females have higher value of iCEB compared to males, which are mean of 4.583 and 3.989 respectively. However, data in the study demonstrated that age has no major influence on the iCEB.

8 $$iCEB = QT/QRS$$

The iCEB increased prior to TdP VT/VF (LQTS) and decreased before non-TdP VT/VF (BS). Assuming the iCEB has no predictive value for both LQTS and BS, an observed result or more extreme result is achieved in less than 0.01% of experiments [31].

### QT Dynamicity

The QT dynamicity represents relationship of QT_i_ and RR_i_ [57], which measured by slope of linear regression of QT_i_/RR_i_, as expressed in Eq. (9). The QT/RR slopes are different in females and males: females have steeper or higher value slopes than males [26, 57].

9 $$QTdynamicity = QT_{i} /RR_{i}$$

The QT dynamicity is a useful predictor for patients with IDCM [26] and AMI [27]. The QT dynamicity on 12-lead ECG is significantly associated with the VT/VF event (P < 0.001). Assuming the QT dynamicity has no predictive value for IDCM patients, an observed result or more extreme result is obtained in less than 0.1% of experiments [26].

## Methods to Improve Detection of the Parameters

Parameters detection is done after preprocessing, i.e. denoise and restructure of ECG signals. However, false detection of the parameters always happens. This may due to misclassification of wide QRS duration as prolonged QT_i_, irregular heart rhythms, as well as slow transition of the signal around T wave end [10, 13]. In order to increase correct detection rate of the parameters identified in Sect. 3, three methods are suggested in this paper.

The first method is averaging, i.e. mean for datasets of parameters. This method is useful to resist fluctuation between the data of each parameter. In this case, Cox [10] recommended to measure the QT_i_ by averaging five or six QT_i_. Similar for TS of HRT, calculation of TS is based on maximum positive regression slope assessed over any five consecutive sinus rhythm RR interval within the first 15 sinus rhythm RR intervals after ventricular premature contraction [25].

The second method is handling of outliers. Presence of outliers, even a very little amount can produce a significant change in the parameters. After identified the outliers, either tossing (removing the outliers) or interpolation (replacing the outliers) can be performed [58]. The definition of outliers may vary among the parameters. For example, there are some recommendation for HRT detection during filtering of RRi sequences. The filtering should include either substantial beat to beat RR intervals difference that are more than 200 ms or substantial difference from an average of five preceding sinus RR intervals that are more than 20%. And, the filtering is recommended to exclude RR intervals that are not in range of 300–2000 ms (outliers) [54].

The third method is application of morphology based algorithms, in which as discussed in Sect. 3. This method is faster than traditional visual assessment (determination of the parameters by experienced ECG signal readers). And, it is especially useful for detection of multiple variations of ECG components such as fQRS and ER. The detailed algorithms are as discussed in [12, 13, 43]. The morphology detection algorithm for fQRS is based on number of identified extrema and notches, point of occurrence of discontinuities, as well as morphology of fragmentation [12]. Whereas, the notched ER and slurred ER patterns are determined using algorithm as in Kentta et al. study [43].

There are also some additional recommended settings in the reviewed literatures for the parameters. For instance, suggested frequency band for LF and HF in HRV are 0.04–0.15 and 0.15–0.4 Hz respectively [28, 55]. Whereas, a higher cutoff frequency of low-pass filter (150 Hz) is needed in order to detect fQRS, else additional spikes within a QRS complex will be masked [36].

## Discussion

As showed in Sect. 2, there are ten ECG parameters have been reviewed, namely QT_c_/QT_v_i/QT_v_i/QT_d_, fQRS, ER, VLP, HRV, HRT, TWA as well as TpTe. These parameters are popular in recent studies and thus investigation regarding to the parameters is necessary.

The aforementioned parameters have achieved significant prediction results in most of the studies; however, some literatures reported that predictive value of the parameters in patients with certain heart diseases are insignificant. From Table 1, it is showed that fQRS have received more attention than other six parameters. The fQRS is a possible new index to identify high-risk patients in arrhythmic events [18], its prognostic role is less known and therefore many studies are done to confirm its predictive value. In the study [59], fQRS in patients with Chagas’s cardiomyopathy are failed to predict the arrhythmic events, although there are many successful evidences in patients with AMI [20], HOCM [19], as well as ARVCM [18]. Similar to fQRS, the VLP is a poor predictor in patients with NSTEMI and ARVCM [15, 17].

Furthermore, most of the reviewed literatures may only reflect the occurrence of VT/VF after a few months or years (long-term) [6–9], but not minutes or hours (short-term). Only HRV has shown short-term predictive value for the arrhythmic event [28, 29]. However, there is another study found no significant association between short-term prediction and parameters such as HRV and QT_vi_ [60]. The conflicting result of HRV as short-term predictor in both studies [28, 60] may due to small sample size and HRV parameters choose in the VAs prediction.

Section 3 has shown there are three main patterns of the parameters that are associated with future VAs, i.e. presence, prolong and decrease. Among these patterns, the presence of the fQRS, ER and VLP can be used for indicating the arrhythmic events. However, the prolongation and decrease of the parameters need a certain range to determine whether the arrhythmia is coming. The ranges that represent the abnormality of the parameters were also discussed in Sect. 3.

The ranges can only be fit after the parameters have been detected and measured. The detection can be missed and the measurement can be inaccurate if lack of appropriate detection or measurement methods. The methods are collected from literatures as in Sect. 4. Both averaging and handling of outliers are suitable for all the parameters. Short signal data is suggested to remove outliers [58]. Whereas long-term signal data might use averaging method to produce better measurement result within a shorter time. For parameters that cannot be measured directly, such as ER and fQRS, morphology based algorithms are helpful to include all of their possible variations. This method is faster than visual interpretation by experienced readers [43].

## Conclusion and Future Works

In conclusion, this study reviewed ten ECG parameters for prediction of VT/VF from 25 articles, as shown in Table 1. Overall, this paper is significant as it achieved three main objectives. Firstly, it provides a way to recognize the patterns of parameters effectively. Using a table as shown in Sect. 3, readers can quickly distinguish the patterns between different parameters prior to occurrence of VAs, i.e. presence, prolongation and reduction. Secondly, it provides a discussion on methods to identify the abnormal ranges of the parameters. The gender and age are factors to influence abnormal ranges of some parameters. Thirdly, this paper describes approaches to improve detection rate of the parameters, as presented in Sect. 4. The approaches are included averaging, outliers handling and application of morphology detection algorithms. In future, ECG parameters for short-term prediction should be pursued. The prediction could be more practical if applied in portable smart devices, such as smartphone.

## References

[1] John RM, Tedrow UB, Koplan BA, Albert CM, Epstein LM, Sweeney MO, Miller AL, Michaud GF, Stevenson WG (2012). Ventricular arrhythmias and sudden cardiac death. Lancet (London, England).

[2] Scapigliati A, Ristagno G, Cavaliere F (2013). The best timing for defibrillation in shockable cardiac arrest. Minerva Anestesiologica.

[3] Fam JM, Ching CK (2011). Review on non-invasive risk stratification of sudden cardiac death. Proceedings of Singapore Healthcare.

[4] Liew R (2011). Electrocardiogram based predictors of sudden cardiac death in patients with coronary artery disease. Clinical Cardiology.

[5] Pietrasik G, Zarba W (2012). QRS fragmentation: Diagnostic and prognostic significance. Cardiology Journal.

[6] Arisha MM, Girerd N, Chauveau S, Bresson D, Scridon A, Bonnefoy E, Chevalier P (2013). In-hospital heart rate turbulence and microvolt T-wave alternans abnormalities for prediction of early life-threatening ventricular arrhythmia after acute myocardial infarction. Annals of Non- Invasive Electrocardiology.

[7] Abdelrahman TM (2014). Prognostic value of T peak-to-end interval for risk stratification after acute myocardial infarction. The Egyptian Journal of Critical Care Medicine.

[8] Aleman-Fernandez, A.A., Dorantes-Sanchez, M., Castro, J., Gonzalez, L.G., Hernandez, Y.C., Marcos, A., & Garcıa, R. (2014). Malignant ventricular arrhythmias in patients with implantable cardioverter-defibrillators: electrical signals which are predictors of recurrence. *CorSalud*, 6(1).

[9] Puzzi MA, Munhoz FP, Carvalho MJ, Gallo LN, Jaid Franca, Lopes RD (2013). The usefulness of T-wave peak to T-wave end interval in identifying malignant arrhythmias in patients with chagas disease. Hellenic Journal of Cardiology.

[10] Cox NK (2011). The QT interval: How long is too long?. Nursing Made Incredibly Easy.

[11] Saleem S, Hussain MM, Majeed SMI, Khan MA (2012). Gender differences of heart rate variability in healthy volunteers. JPMA-Journal of the Pakistan Medical Association.

[12] Maheshwari S, Acharyya A, Puddu PE, Mazomenos EB, Leekha G, Maharatna K, Schiariti M (2013). An automated algorithm for online detection of fragmented QRS and identification of its various morphologies. Journal of the Royal Society, Interface.

[13] Madeiro JPV, Nicolson WB, Cortez PC, Marques JAL, Vazquez- Seisdedos CR, Elangovan N, Ng GA, Schlindwein FS (2013). New approach for T-wave peak detection and T-wave end location in 12-lead paced ECG signals based on a mathematical model. Medical Engineering & Physics.

[14] Huang Z, Patel C, Li W, Xie Q, Wu R, Zhang L, Tang R, Wan X, Ma Y, Zhen W, Gao L, Yan GX (2009). Role of signal-averaged electrocardiograms in arrhythmic risk stratification of patients with Brugada syn- drome: a prospective study. Heart Rhythm: The Official Journal of the Heart Rhythm Society.

[15] Abe A, Kobayashi K, Yuzawa H, Sato H, Fukunaga S, Fujino T, Okano Y, Yamazaki J, Miwa Y, Yoshino H (2012). Comparison of late potentials for 24 hours between Brugada syndrome and arrhythmogenic right ventricular cardiomyopathy using a novel signal-averaging system based on Holter ECG. Circulation: Arrhythmia and Electrophysiology.

[16] Patel RB, Ng J, Reddy V, Chokshi M, Parikh K, Subacius H, Alsheikh- Ali AA, Nguyen T, Link MS, Goldberger JJ (2010). Early repolarization associated with ventricular arrhythmias in patients with chronic coronary artery disease. Circulation: Arrhythmia and Electrophysiology.

[17] Wang J, Sui XT, Sun YX, Li Y, Yang G, Xu F, Zhang YL, Zhang XG (2013). Differences of ventricular late potential between acute STEMI and NSTEMI patients. West Indian Medical Journal.

[18] Canpolat U, Kabakci G, Aytemir K, Dural M, Sahiner L, Yorgun H, Sunman H, Kaya EB, Tokgozoglu L, Oto A (2013). Fragmented QRS complex predicts the arrhythmic events in patients with arrhythmogenic right ventricular cardiomyopathy/dysplasia. Journal of Cardiovascular Electrophysiology.

[19] Femenia F, Arce M, Van Grieken J, Trucco E, Mont L, Abello M, Merino JL, Rivero-Ayerza M, Gorenek B, Rodriguez C, Hopman WM, Baranchuk A (2013). Fragmented QRS as a predictor of arrhyth- mic events in patients with hypertrophic obstructive cardiomyopathy. Journal of Interventional Cardiac Electrophysiology.

[20] Ma SY, Lv JL, Liu ZB, Li ZP, Wang LX (2013). Relationship between fragmented QRS complex and ventricular arrhythmias in patients with a previous myocardial infarction. Experimental & Clinical Cardiology.

[21] Sha J, Zhang S, Tang M, Chen KP, Zhao XR, Wang FZ (2011). Fragmented QRS is associated with All-cause mortality and ventricular arrhythmias in patient with idiopathic dilated cardiomyopathy. Annals of Noninvasive Electrocardiology.

[22] Haıssaguerre M, Derval N, Sacher F, Jesel L, Deisenhofer I, de Roy L, Pasquie JL, Nogami A, Babuty D, Yli-Mayry S (2008). Sudden cardiac arrest associated with early repolarization. New England Journal of Medicine.

[23] Tereshchenko LG, Fetics BJ, Domitrovich PP, Lindsay BD, Berger RD (2009). Prediction of ventricular tachyarrhythmias by intracardiac repolarization variability analysis. Circulation-Arrhythmia and Electro- physiology.

[24] Wahab A, Alvi S, Panwar BR, Budania S (2012). A study of QT dispersion as a prognostic indicator in acute myocardial infarction. Int Cardiovascular Res Journal.

[25] Li-na R, Xin-hui F, Li-dong R, Jian G, Yong-quan W, Guo-xian Q (2012). Ambulatory ECG-based T-wave alternans and heart rate turbulence can predict cardiac mortality in patients with myocardial infarction with or without diabetes mellitus. Cardiovascular diabetology.

[26] Iacoviello M, Forleo C, Guida P, Romito R, Sorgente A, Sorrentino S, Catucci S, Mastropasqua F, Pitzalis M (2007). Ventricular repolarization dynamicity provides independent prognostic information toward major arrhythmic events in patients with idiopathic dilated cardiomyopathy. Journal of the American College of Cardiology.

[27] Chen X, Hu Y, Fetics BJ, Berger RD, Trayanova NA (2011). Unstable QT interval dynamics precedes ventricular tachycardia onset in patients with acute myocardial infarction: A novel approach to detect instability in QT interval dynamics from clinical ECG. Circulation: Arrhythmia and Electrophysiology.

[28] Ebrahimzadeh E, Pooyan M, Bijar A (2014). A novel approach to Predict sudden cardiac death (SCD) using nonlinear and time-frequency analyses from HRV signals. PLoS ONE.

[29] Rozen G, Kobo R, Beinart R, Feldman S, Sapunar M, Luria D, Eldar M, Levitan J, Glikson M (2013). Multipole analysis of heart rate variability as a predictor of imminent ventricular arrhythmias in ICD patients. Pacing and Clinical Electrophysiology.

[30] Lu HR, Yan GX, Gallacher DJ (2013). A new biomarker index of cardiac electrophysiological balance (iCEB)—plays an important role in drug-induced cardiac arrhythmias: Beyond QT-prolongation and Torsades de Pointes (TdPs). Journal of Pharmacological and Toxico- logical Methods.

[31] Robyns T, Lu HR, Gallacher DJ, Garweg C, Ector J, Willems R, Janssens S, Nuyens D (2016). Evaluation of index of cardio- electrophysiological balance (iCEB) as a new biomarker for the identification of patients at increased arrhythmic risk. Annals of Nonin-vasive Electrocardiology.

[32] Steyerberg E (2009). Clinical Usefulness. Clinical prediction models.

[33] Alturki A (2015). SM Gr up the value of P value in the medical SM. Journal of Public Health and Epidemiology.

[34] Gadaleta M, Giorgio A (2012). A method for ventricular late potentials detection using time-frequency representation and wavelet denoising. ISRN Cardiology.

[35] Tsutsumi T, Takano N, Matsuyama N, Higashi Y, Iwasawa K, Nakajima T (2011). High-frequency powers hidden within QRS complex as an additional predictor of lethal ventricular arrhythmias to ventricular late potential in post myocardial infarction patients. Heart Rhythm.

[36] Take Y, Morita H (2012). Fragmented QRS: What is the meaning?. Indian Pacing and Electrophysiology Journal.

[37] Das MK, Zipes DP (2010). Role of the fragmented QRS complexes on a routine 12-lead ECG in predicting mortality and sudden cardiac death. Rev Argent Cardiology.

[38] Derval N, Shah A, Jaıs P (2011). Definition of early repolarization a tug of war. Circulation.

[39] Smith, S.W., Khalil, A., Henry, T.D., Rosas, M., Chang, R.J., Heller, K., Scharrer, E., Ghorashi, M., & Pearce, L.A. (2012). Electrocardiographic differentiation of early repolarization from subtle anterior ST-segment elevation myocardial infarction. *Annals of Emergency Medicine*, 60(1), 45–56. e2. 10.1016/j.annemergmed.2012.02.015 22520989

[40] Bastiaenen R, Behr ER (2012). Benign or malignant, early or delayed: The changing face of early repolarization. Europace.

[41] Aizawa Y, Sato A, Watanabe H, Chinushi M, Furushima H, Horie M, Kaneko Y, Imaizumi T, Okubo K, Watanabe I, Shinozaki T, Aizawa Y, Fukuda K, Joo K, Haissaguerre M (2012). Dynamicity of the J-wave in idiopathic ventricular fibrillation with a special reference to pause- dependent augmentation of the J-wave. Journal of the American College of Cardiology.

[42] Adler A, Rosso R, Viskin D, Halkin A, Viskin S (2013). What do we know about the “malignant form” of early repolarization?. Journal of the American College of Cardiology.

[43] Kentta T, Porthan K, Tikkanen JT, Vaananen H, Oikarinen L, Viitasalo M, Karanko H, Laaksonen M, Huikuri HV (2014). Sensitivity and specificity of automated detection of early repolarization in standard 12 lead electrocardiography. Annals of Noninvasive Electrocardiology.

[44] Letsas, K.P., Weber, R., Astheimer, K., Kalusche, D., & Arentz, T. (2009). Tpeak-Tend interval and TpeakTend/QT ratio as markers of ventricular tachycardia inducibility in subjects with Brugada ECG phenotype. *Europace*, 6, eup357. 10.1093/europace/eup357 19897501

[45] Hetland M, Haugaa KH, Sarvari SI, Erikssen G, Kongsgaard E, Edvardsen T (2014). A novel ECG-index for prediction of ventricular arrhythmias in patients after myocardial infarction. Annals of Noninvasive Electrocardiology.

[46] Sun X, Cai J, Fan X, Han P, Xie Y, Chen J, Xiao Y, Kang YJ (2013). Decreases in electrocardiographic R-wave amplitude and QT interval predict myocardial ischemic infarction in rhesus monkeys with left anterior descending artery ligation. PLoS ONE.

[47] Dobson CP, Kim A, Haigney M (2013). QT variability index. Progress in Cardiovascular Diseases.

[48] Piccirillo G, Magri D, Matera S, Magnanti M, Torrini A, Pasquazzi E, Schifano E, Velitti S, Marigliano V, Quaglione R, Barilla F (2007). QT variabil- ity strongly predicts sudden cardiac death in asymptomatic subjects with mild or moderate left ventricular systolic dysfunction: A prospective study. European Heart Journal.

[49] Quan XQ, Zhou HL, Ruan L, Lv JG, Yao JH, Yao F, Huang K, Zhang CT (2014). Ability of ambulatory ECG-based T-wave alternans to modify risk assessment of cardiac events: A systematic review. Bmc Cardiovascular Disorders.

[50] Nieminen T, Verrier RL (2010). Usefulness of T-wave alternans in sudden death risk stratification and guiding medical therapy. Annals of Noninvasive Electrocardiology.

[51] Monasterio V, Laguna P, Cygankiewicz I, Martinez JP (2011). Average T- wave alternans activity in ambulatory ECGs. Computing in Cardiology.

[52] Verrier RL, Klingenheben T, Malik M, El-Sherif N, Exner DV, Hohnloser SH, Ikeda T, Martınez JP, Narayan SM, Nieminen T, Rosenbaum DS (2011). Microvolt T-wave alternans physiological basis, methods of measurement, and clinical utility consensus guideline by International Society for Holter and Noninvasive Electrocardiology. Journal of the American College of Cardiology.

[53] Chen DH, Yang S (2009). The impact of frequency aliasing on spectral method of measuring T wave alternans. Journal of Biomedical Science and Engineering.

[54] Bauer A, Malik M, Schmidt G, Barthel P, Bonnemeier H, Cygankiewicz I, Guzik P, Lombardi F, Muller A, Oto A, Schneider R, Watanabe M, Wichterle D, Zareba W (2008). Heart rate turbulence: Standards of measurement, physiological interpretation, and clinical Use: International Society for Holter and Noninvasive Electrophysiology Consensus. Journal of the American College of Cardiology.

[55] Shaik, N.A., & Ramdas, D. (2014). Empirical mode decomposition for frequency analysis of heart rate variability. In *Electronics and Communication Systems (ICECS), 2014 International Conference on* (pp 1–6). doi:10.1109/ECS.2014.6892628.

[56] Saleem S, Hussain MM, Majeed SMI, Khan MA (2012). Gender differences of heart rate variability in healthy volunteers. JPMA-Journal of the Pakistan Medical Association.

[57] SisAkova M, Toman O, Florianova A, Vit P, Gaillyova R, Kadlecova J, Chroust K, Papousek I, Spinar J (2005). Analysis of Qt dynamicity behaviour in relationship to the risk of sudden cardiac death. A pilot study. Scripta Med- Ica (BRNO).

[58] Pradhan L, Islam M (2010). Replacing outliers with existing data in inter beat interval signal for heart rate variability analysis. IBIs.

[59] Baranchuk A, Femenia F, LopezDiez JC, Muratore C, Valentino M, Retyk E, Galizio N, Toro D, Alonso K, Hopman WM (2014). Fragmented surface ECG was a poor predictor of appropriate therapies in patients with Chagas cardiomyopathy and ICD implantation (Fragmented ECG in Chagas Cardiomyopathy Study). Annals of Noninvasive Electrocardiology.

[60] Sachdev M, Fetics BJ, Lai S, Dalal D, Insel J, Berger RD (2010). Failure in short-term prediction of ventricular tachycardia and ventricular fibrillation from continuous electrocardiogram in intensive care unit patients. Journal of Electrocardiology.

